# A New Case Report of Traboulsi Syndrome: A Literature Review and Insights Into Genotype–Phenotype Correlations

**DOI:** 10.3390/genes15091120

**Published:** 2024-08-25

**Authors:** Marisol Ibarra-Ramírez, Luis D. Campos-Acevedo, Aristides Valenzuela-Lopez, Luis Arturo López-Villanueva, Marissa Fernandez-de-Luna, Jibran Mohamed-Noriega

**Affiliations:** 1 Department of Genetics, University Hospital and Faculty of Medicine, Autonomous University of Nuevo Leon (UANL), Monterrey 64460, Mexico; mibarrar@uanl.edu.mx (M.I.-R.); lcamposa@uanl.edu.mx (L.D.C.-A.); 2 Department of Ophthalmology, University Hospital and Faculty of Medicine, Autonomous University of Nuevo Leon (UANL), Monterrey 64460, Mexico; aristides.valenzuelalp@uanl.edu.mx (A.V.-L.); luis.lopezvnv@uanl.edu.mx (L.A.L.-V.); mfernandez.109881@uanl.edu.mx (M.F.-d.L.)

**Keywords:** Traboulsi syndrome, ASPH gene, lens subluxation, genetic variability, spontaneous blebs

## Abstract

Traboulsi syndrome is a rare genetic disorder characterized by facial dysmorphism, lens subluxation, anterior segment anomalies, and spontaneous filtering blebs. The syndrome is due to mutations in the *ASPH* gene, which plays a crucial role in the development and maintenance of the lens. This case report describes the clinical and genetic findings in a Mexican male with Traboulsi syndrome, highlighting the identification of a novel *ASPH* variant. A 21-year-old male presented with trauma to the right eye while playing soccer. He had a history of lens subluxation and dysmorphic facial features. Ophthalmic examination revealed right eye lens subluxation into the anterior chamber (with signs of a previous episode of acute angle closure) and left eye posterior and inferior lens subluxation with sectorial iris atrophy. Genetic analysis identified a pathogenic *ASPH* variant (NM_004318.3:c.1892G>A, p.Trp631*) and a novel likely pathogenic variant (deletion of exons 20–21), confirming Traboulsi syndrome. This is the first instance of Traboulsi syndrome in the Mexican population. The absence of spontaneous filtering blebs in this patient supports previous reports of the wide phenotypic variability that could be related to the type of mutation. This novel *ASPH* variant expands the known genetic heterogeneity of Traboulsi syndrome.

## 1. Introduction

Traboulsi syndrome, also known as FDLAB (Facial Dysmorphism, Lens subluxation, Anterior segment anomalies, and spontaneous filtering Blebs), is an exceedingly rare genetic syndrome first described in 1995 within a Druze community in Lebanon [1]. Initially considered an autosomal recessive disease, subsequent cases within the same community supported this inheritance pattern [2]. The ASPH gene was identified as the cause of Traboulsi syndrome in 2014; it encodes aspartyl/asparaginyl β-hydroxylase, junctin, and junctate [3,4]. The ASPH enzyme is responsible for the hydroxylation of aspartic acid and asparagine residues in proteins containing epidermal growth factor (EGF) domains. It has been previously proposed [3] that mutations in genes affecting the interaction with the EGF domain (which are highly expressed during lens development) are the mechanism behind lenticular abnormalities in Traboulsi syndrome, as virtually all genes implicated in lens subluxation (*FBN1*, *ADAMTSL4*, *ADAMTS10*, and *ADAMTS17*) encode proteins that interact with EGF [3]. 

Various cases with variable expressivity have been reported [5,6,7,8,9,10,11,12,13,14]. Recent reports have also noted cardiovascular involvement [15,16]. Limited ASPH variants have been identified without clear genotype–phenotype correlations. Here, we present a male with Traboulsi syndrome with a milder ophthalmic phenotype and no spontaneous filtering blebs, and report the identification of one pathogenic and one novel *ASPH* variant. A literature review was conducted to explore the potential genotype–phenotype correlation in patients with Traboulsi syndrome.

## 2. Material and Methods

### Case Description

A 21-year-old male attended ophthalmology accident and emergency (A&E) after experiencing trauma to the right eye (OD) while playing soccer. He was born after cesarean section at 41 gestational weeks, with normal weight and height, to non-consanguineous parents. During childhood, the patient complained of blurred vision and was diagnosed with lens subluxation and dysmorphic facial features but never underwent genetic testing. 

**Figure 1:**
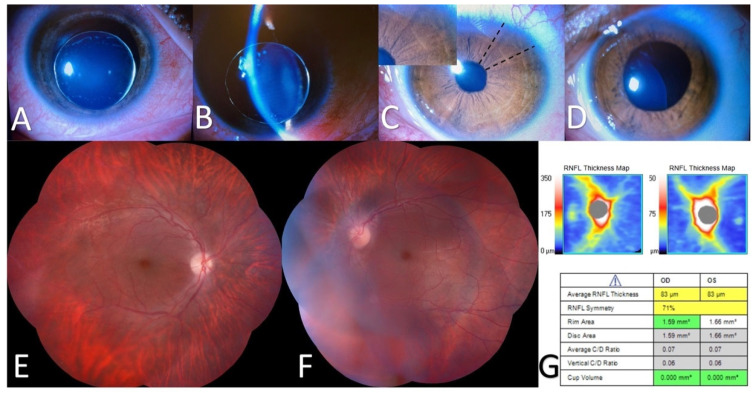
(**A**) Anterior segment photograph of right eye with clear cornea, iris atrophy, glaukomflecken, and anterior microspherical lens subluxation depicted with a slit lamp photograph in (**B**). (**C**) Anterior segment photograph of left eye with clear cornea, sectorial iris atrophy (area between dashed lines and upper left box) and posterior inferior microspherical lens subluxation best seen under pupillary dilation in (**D**). (**E**,**F**) Fundus photograph in right and left eye: small and slightly pale discs and tessellated fundus appearance. (**G**) Optic nerve OCT with reduced RNFL thickness and small discs in both eyes.

The initial ophthalmic examination at A&E revealed an uncorrected visual acuity of counting fingers that improved to 20/400 with a pinhole in both eyes. The OD had periorbital edema, conjunctival hyperemia, a clear cornea, and lens subluxation into the anterior chamber (Figure 1). In addition, the OD had signs of a previous pupillary block such as iris atrophy and glaukomflecken (scattered white dots in the anterior lens capsule). The left eye (OS) had a clear cornea, iridodonesis, sectorial iris atrophy, and posterior and inferior lens subluxation. A fundus examination in both eyes revealed small and slightly pale discs with a cup disc ratio of 0.1, and a tessellated fundus appearance. The endothelial cell density was 1377 cells/mm^2^ in OD and 3448 cells/mm^2^ in LE. The patient underwent an uncomplicated clear lens extraction via pars plana, a posterior vitrectomy, and peripheral photocoagulation to peripheral retinal tears identified during surgery. Three years after surgery, the best-corrected visual acuity was 20/30 in both eyes using aphakic contact lenses, no corneal edema was present, the intraocular pressure (IOP) was 10 in both eyes without treatment, and there were no areas of scleral thinning or filtering blebs.

A clinical geneticist referral examination revealed a weight of 82 kg, a height of 168 cm, a head circumference of 57.3 cm, an apparent age older than chronological, a normocephalic, elongated face, downward slanting palpebral fissures, a prominent nose, nasal alae hypoplasia, crowded teeth, a high-arched palate, midface hypoplasia, micrognathia, eutrophic and symmetric limbs, and dorsal-level scoliosis.

## 3. Results

**Figure 2:**
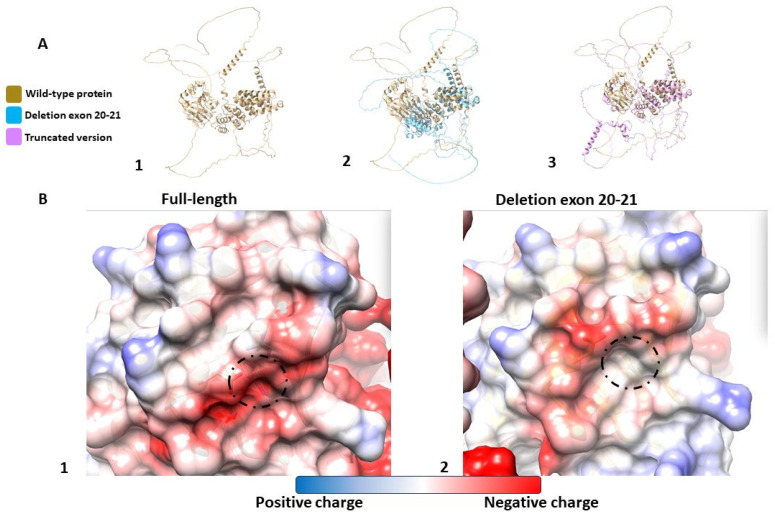
(**A**) Three-dimensional model created by AlphaFold of the wild-type protein (**A1**) compared to the protein with the deletion of exons 20–21 (**A2**) and the truncated protein (**A3**). (**B**) Three-dimensional model of the electrostatic potential created by AlphaFold of the wild-type protein (**B1**) and the protein with deletion exons 20–21 (**B2**).

Given the clinical suspicion of connective tissue disorders, a comprehensive analysis was conducted using the parallel massive sequencing of a multigene panel on an Illumina platform 5.8. This analysis revealed a pathogenic variant in the *ASPH* gene, NM_004318.3:c.1892G>A (p.Trp631*), and a novel, likely pathogenic, variant characterized by the deletion of exons 20–21, confirming a compound heterozygous state consistent with Traboulsi Syndrome. The carrier status was established in both parents through targeted variant analysis. To investigate the role of the novel variant in the protein, a comparative analysis was performed on the wild-type protein and its mutated form, as predicted by AlphaFold2 and Alphafold2-multimer. (Figure 2).

## 4. Discussion

Traboulsi syndrome is a rare genetic disorder characterized by a combination of ocular and craniofacial abnormalities that should be considered in the differential diagnosis of Marfan and Weill–Marchesani syndromes. In this case report, we describe a 21-year-old Mexican male with Traboulsi syndrome, a condition not previously reported in the Mexican population. Our comprehensive evaluation of this patient’s clinical presentation and genetic profile provides valuable insights into the diverse genetic basis of lens subluxation and underscores the importance of genetic testing in diagnosing rare genetic disorders. Timely genetic testing is particularly useful in detecting syndromes associated with diverse phenotypes, such as Traboulsi syndrome, which might present to an ophthalmologist with low vision due to lens subluxation or pain secondary to ocular hypotony in patients with spontaneous blebs.

### Phenotypic Manifestations and Ophthalmologic Complications

Spontaneous blebs were originally reported as a hallmark of Traboulsi syndrome [10]. However, the current and previous reports highlight the importance of considering this syndrome in the differential diagnosis of lens subluxation. In the current case, spontaneous blebs did appear three years after surgery; however, it is important to mention that surgical techniques have evolved since the first descriptions of this syndrome in 1995, but even with the use of small-gauge trocar-cannula systems, all sclerotomies had to be sutured tightly to prevent leaks and hypotony [1]. The absence of spontaneous or postoperative blebs in the present case is probably related to the variability in phenotypes that could be associated with specific genetic variants. In cases with spontaneous blebs, it is possible that even tightly sutured sclerotomies could have triggered the formation of new blebs or scleral thinning.

The ocular manifestations in the current case revealed significant differences between the right and left eyes, a common feature in Traboulsi syndrome. The right eye exhibited the luxation of the lens into the anterior chamber, leading to a pupillary block. In contrast, the left eye displayed posterior and inferior lens subluxation, sectorial iris atrophy, and iridodonesis. The fundus examination revealed small discs with a tessellated appearance, consistent with previous reports in Traboulsi syndrome patients. The diversity of phenotypes in Traboulsi syndrome could be caused by genotype–phenotype differences and/or the distinct times in the natural history of the disease that the patients were investigated at and reported. For instance, the axial length and presence of myopia or glaucoma vary significantly between publications in part due to the inclusion of patients with or without filtering blebs that change the IOP and axial length, or the inclusion of patients with or without lens subluxation that change the refraction and myopia status.

## 5. Genetic Insights and Variability

Genetic evaluation played a pivotal role in confirming the diagnosis of Traboulsi syndrome in this patient. The first variant (NM_004318.3:c.1892G>A, p.Trp631*) was classified as pathogenic, providing strong evidence for its contribution to the development of Traboulsi syndrome in this patient. The second variant, a novel deletion involving exons 20–21, was considered likely pathogenic, suggesting its potential involvement in the syndrome’s pathogenesis. Importantly, these variants have not been reported in the existing literature, adding to the genetic heterogeneity of Traboulsi syndrome.

The complications during Traboulsi syndrome most commonly reported in the literature are bleb formation and lens dislocation [3,10]. Our analysis aimed to assess the frequency of these manifestations in relation to specific genetic variants. Among the 28 documented cases, our comprehensive review, incorporating molecular analysis, revealed 17 distinct genetic variants: five missense, eight nonsense, two splicing site mutations, one large deletion, and one synonymous variant (Table 1). Four cases were compound heterozygous. Interestingly, 85.7% of patients with missense mutations develop blebs, compared to only 33% of patients with nonsense variants. This correlation between the variant type and the risk of bleb formation warrants a further detailed investigation of the genotype–phenotype relationship, which has not been well-characterized due to the limited number of reported cases. We also analyzed other characteristics of previously reported cases and the types of variants without finding any association that suggests a stronger genotype–phenotype correlation. However, the main limitation of this review is the small number of patients and the lack of detailed ophthalmological complication reports in all cases.

Interestingly, most variants are located in exons 21–25, involving the AspH oxygenase domain; this domain is essential for productive catalysis as it interacts with the epidermal growth factor-like domain (EGFD) substrates of AspH (Pfeffer et al., 2019). Aspartyl β-hydroxylase (ASPH) is an enzyme featuring a C-terminal catalytic domain that plays a pivotal role in catalyzing the posttranslational hydroxylation of aspartic acid or asparagine residues within EGFDs of various proteins. A detailed comparative analysis of the wild-type protein and its mutated counterpart was conducted, as modelled by AlphaFold. The analysis highlighted structural differences between the wild-type and mutated proteins. Notably, a shift in polarity was observed in the new variant. This alteration likely affects the binding site, which is integral to its function, as previously suggested by Patel et al. [3]. Such a modification could be directly linked to the protein’s malfunction, offering a potential explanation for the phenotypic manifestations observed in individuals with the mutation.

ASPH plays a vital role in human development by hydroxylating aspartic acid and asparagine residues on proteins that contain epidermal growth factor domains. This post-translational modification is critical for the proper function of these proteins involved in numerous developmental pathways. Dinchuk et al., in 2002, explored the consequences of disrupting the catalytic domain of ASPH in mice while keeping the coding regions of junctin and junctate unaffected. ASPH-null mice exhibited a spectrum of developmental defects, including syndactyly, facial dysmorphology, and a mild defect in hard palate formation. Notably, these developmental anomalies resembled those observed in knockouts and hypomorphs of the Notch ligand Serrate-2 (*JAG2*; 602570). This striking similarity suggests a potential interplay between the aspartyl β-hydroxylation of epidermal growth factor domains and the modulation of the Notch signaling pathway. These findings underscore the significance of ASPH in orchestrating critical developmental processes [3].

The mutations identified in our study are likely to impair the enzymatic function of ASPH, disrupting this crucial hydroxylation process [5]. The resultant loss of function could lead to the developmental anomalies associated with ASPH-related disorders, such as those observed in Traboulsi syndrome.

The genes *FBN1, ADAMTSL4*, *ADAMTS10*, and *ADAMTS17* play crucial roles in the structural integrity and function of the eye’s lens. *FBN1* encodes fibrillin-1, a protein essential for the formation of elastic fibers in connective tissue, which contributes to the stability and positioning of the lens. Mutations in *FBN1* are commonly associated with Marfan syndrome and related conditions that feature lens dislocation [18]. *ADAMTSL4* encodes a member of the ADAMTS-like protein family, which is involved in the assembly of the extracellular matrix; mutations in this gene lead to autosomal recessive isolated lens subluxation [19]. *ADAMTS10* and *ADAMTS17* encode metalloproteases that participate in the processing of extracellular matrix components, crucial for maintaining lens zonule fibers [20]. Mutations in these genes disrupt the extracellular matrix’s normal structure, resulting in lens subluxation. Collectively, these genes encode proteins that interact with epidermal growth factor domains, highlighting the importance of proper EGFD function in maintaining lens stability and preventing dislocation.

## 6. Conclusions

In conclusion, this case report not only presents the first documented case of Traboulsi syndrome in the Mexican population, but also provides valuable insights into the clinical and genetic aspects of this rare genetic disorder. The identification of pathogenic and novel *ASPH* gene variants expands our understanding of the genetic basis of Traboulsi syndrome and its variability. Further research is warranted to elucidate the functional consequences of these variants in in vitro studies and explore potential therapeutic strategies for managing this challenging condition.

## Figures and Tables

**Table 1:** Reported cases of Traboulsi syndrome in the literature. It shows the different genetic variants described in the literature and whether an association with blebs and/or lens dislocation was reported. Deletion entire coding region of the gene. ^ These variants were reported by Patel in 2014 from cases reported by Haddad and Mansour.

Reference	Variants	Type of Mutation	Exon	Spontaneous Blebs	Lens Subluxation
Abarca Barriga 2018 [5]	c.171G>A p.W57*	Nonsense	Exon 2	+	+
Jones 2022 [15]	c.1497_1500delGGCA p. Lys499Asnfs*12	Nonsense	Exon 19	−	−
Jones 2022 [15]	c.1497_1500delGGCA p. Lys499Asnfs*12	Nonsense	Exon 19	−	+
Jones 2022 [15]	c.1626G>A, p. Glu542=	Synonym	Exon 20	−	+
Musleh 2023 [14]	c.1695 C>A p. Tyr565*	Nonsense	Exon 21	−	+
Kulkarni 2019 [7]	c.1695C>A p.Tyr565*	Nonsense	Exon 21	−	+
Kulkarni 2019 [7]	c.1695C>A p.Tyr565*	Nonsense	Exon 21	−	+
Senthill 2020 [11]	c.1853 T>A p.Leu618Gln	Missense	Exon 21	+	+
Senthill 2020 [11]	c.1853 T>A p.Leu618Gln	Missense	Exon 21	+	+
Patel 2014 [3]	c.1852_1856delinsGGG p. Ser589Glufs*18	Nonsense	Exon 22	+	+
Chandran 2019 [6]	c.1869_1873 del GGACT p.Asp624Glufs*13	Nonsense	Exon 22	+	+
Lei 2020 [16]	c.1910delA/ p. Asn637Metfs*15	Nonsense	Exon 23	−	+
Kulkarni 2019 [7]	c.2127-2delA p.?	Splincing	Exon 24	−	+
Shanmugam 2020 [8]	c. 2062 C>G p.Arg688Gln	Missense	Exon 24	+	−
Jones 2022 [15]	c.2062C>T p. Arg688*	Nonsense	Exon 24	+	+
Haddad 2001 [2] ^	c.2203C>T p. Arg735Trp.	Missense	Exon 25	−	−
Mansour 2013 [17] ^	c.2203C>T p. Arg735Trp.	Missense	Exon 25	+	+
Senthill 2020 [11]	c.2246 G>A p.Ser749Asn	Missense	Exon 25	+	+
Senthill 2020 [11]	c.2204 G>A p.Arg735Glu	Missense	Exon 25	+	+
Van Hoorde 2021 [10]	c.2181_2183dup p. Val727_Trp728ins*	Nonsense	Exon 25	−	+
Van Hoorde 2021 [10]	c.2181_2183dup, p.Val727_Trp728ins*	Nonsense	Exon 25	+	+
Jones 2022 [15]	c.2181_2183dupATG p. Trp728*	Nonsense	Exon 25	−	+
Jones 2022 [15]	c.2181_2183dupATG p. Trp728*	Nonsense	Exon 25	−	+
Jones 2022 [15]	c.2181_2183dupATG p. Trp728*	Nonsense	Exon 25	−	+
Lima 2023 [13]	c.1765-1G>A p. ?	Splincing	Intron 21	+	−
Kulkarni 2019 [7]	c.2127-2delA p.?	Splincing	Intron 24	−	+
Musleh 2023 [14]	c. 2127-2delA	Splincing	Intron 24	−	+

## Data Availability

The raw data supporting the conclusions of this article will be made available by the authors on request.
